# Individual and household factors associated with ownership of long-lasting insecticidal nets and malaria infection in south-central Ethiopia: a case–control study

**DOI:** 10.1186/s12936-017-2048-9

**Published:** 2017-10-06

**Authors:** Wakgari Deressa

**Affiliations:** Department of Preventive Medicine, School of Public Health, College of Health Sciences, Addis Ababa University, P. O. Box 9086, Addis Ababa, Ethiopia

**Keywords:** Case–control, Long-lasting insecticidal net, Malaria, Ownership, Oromia, Ethiopia

## Abstract

**Background:**

A recent considerable decline in malaria morbidity and mortality in Ethiopia is likely to be followed by changes in the practice of effective preventive measures and malaria risk factors. This study aimed to identify determinants of long-lasting insecticidal nets (LLINs) ownership and risk of malaria infection.

**Methods:**

A matched case–control study of 191 case and 377 control households was conducted between October 2014 and November 2015 in Adami Tullu district in south-central Ethiopia. Cases were microscopy or rapid diagnostic test confirmed malaria patients identified at three health centers and nine health posts, and matched on age with two neighbourhood controls. Information was collected on socio-demographic factors, house structure, knowledge on malaria and ownership of LLINs. The logistic regression model was used to determine predictors of LLINs ownership and malaria infection.

**Results:**

All cases were infections due to either *Plasmodium falciparum* (71.2%) or *Plasmodium vivax* (28.8%). About 31% of the study households had at least one LLINs. Significant determinants of LLINs ownership were household’s head malaria knowledge [adjusted odds ratio (AOR) = 2.47, 95% confidence interval (CI) 1.44–4.22], educational status [read and write (AOR = 6.88, 95% CI 2.30–20.55), primary education or higher (AOR = 5.40, 95% CI 1.57–18.55)], farmer respondent (AOR = 0.35, 95% CI 0.17–0.76), having ≥ 3 sleeping areas (AOR = 6.71, 95% CI 2.40–18.77) and corrugated roof type (AOR = 2.49, 95% CI 1.36–4.58). This study was unable to identify important risk factors of malaria infection with regard to sex, household wealth index, house structure, ownership of LLINs, keeping livestock inside house, staying overnight outdoor or having malaria during the last 6 months.

**Conclusions:**

Household socio-economic status, educational status and knowledge on malaria were important predictors of LLINs ownership. Households with farmer respondents were less likely to own LLINs. Addressing these factors could improve household’s ownership of LLINs. The importance of factors associated with malaria infection was less evident in the current low transmission setting, and necessitates further epidemiological study.

## Background

A considerable decline in malaria morbidity and mortality in Ethiopia has been observed since 2005 [1, 2]. Scaling-up of effective anti-malarial interventions such as long-lasting insecticidal nets (LLINs), indoor residual spraying (IRS) and case management using artemether–lumefantrine (AL, Coartem^®^) are the main reasons for the decline [3]. According to the Ethiopia Malaria Indicator Surveys (EMIS), malaria prevalence was low and estimated at 0.9% in 2007, 1.3% in 2011 and 0.5% in 2015 [4–6]. Nevertheless, malaria remains one of the 10 leading public health problems in the country, accounting for 3.5% of outpatient consultations, 2% of hospital admissions and 1% of inpatient deaths in 2014/2015 [7].

Ownership of LLIN and its proper use by the community is one of the key determinants for the success of malaria intervention. However, household’s ownership and utilization of LLINs has been low in Ethiopia despite efforts made to improve its universal coverage. The EMIS showed that the proportion of ownership of at least one LLIN was 66% in 2007, 55% in 2011 and 64% in 2015 [4–6]. Several studies on community acceptance and use of LLINs in Ethiopia have revealed that various cultural, behavioural and socio-economic factors influence ownership and use of LLINs [8–10].

Various environmental, household and individual factors are associated with malaria risk. Studies reported that housing types and domestic animals staying in the house [11], number of LLINs in the household and use of preventive methods [12, 13], age of individuals and houses with holes [14] and household wealth status [12] were significantly associated with malaria risk.

Ethiopia has adopted malaria elimination strategy and the target to eliminate the disease in selected low transmission areas was set for 2020 [15]. The strategy requires a reliable evidence to improve understanding of malaria risk factors to inform proper targeting of anti-malarial interventions. As a result, understanding individual and household factors that determine malaria incidence and use of effective preventive measures such as LLINs and IRS is very crucial.

However, the conventional community-based cross-sectional surveys, such as malaria indicator surveys or prospective cohort study designs, would not be an efficient way to identify malaria cases or to assess risk factors because of the very large number of participants needed to be recruited. To study rare infections or diseases, the case–control methods are often used in identifying risk factors, but have been less frequently applied in malaria research during the last decade. This matched case–control study was undertaken in a predominantly rural population in south-central Ethiopia to investigate individual and household factors associated with LLINs ownership and malaria infection.

## Methods

### Study setting and population

The study was conducted in Oromia Regional State in Ethiopia in “Adami Tullu Jiddo Kombolcha” district, commonly known as “Adami Tullu” district. The district is located in the Great Rift Valley, with a projected population size of 173,000 people for the 2014 based on the 2007 census [16]. It is administratively divided into 48 *kebeles*, the smallest of local government. A *kebele* is further divided territorially into the *garee* (team or group), comprised of 40–90 households. The district is characterized by a low annual rainfall, with the total annual precipitation of 700 mm that peaks to 250 mm during July and August [17]. The average minimum and maximum annual temperature is 14.5 to 27.7 °C, respectively. The main ethnic group inhabiting the area is Oromo, and the majority of the population lives in rural areas. The economy of the area primarily depends on subsistence farming and livestock rearing. In 2014, there were one hospital, nine health centres and 43 health posts in the district. Each *kebele* is intended to have one health post staffed with two Health Extension Workers (HEWs). There were also a number of private health facilities serving the community.

Malaria is the leading cause of morbidity and mortality in the district, transmission being peaking shortly after the end of the rainy season during July–August. Transmission is seasonal, and characterized with several epidemics recorded in the past [18]. Both *Plasmodium falciparum* and *Plasmodium vivax* account for almost all of the infections, while *Anopheles arabiensis* is the dominant malaria vector in the area [19, 20]. The use of LLINs and IRS in addition to malaria case treatment has been the major malaria prevention and control tools used in the area. A free distribution of LLINs by the District Health Office undertakes 2–3 years to all households in malarious localities, and IRS operation undertakes annually during July–August in the targeted *kebeles*. Malaria diagnosis and treatment is provided free-of-charge in the public sector. Artemether–lumefantrine and chloroquine are the first-line anti-malarial drugs for the treatment of uncomplicated falciparum and vivax malaria, respectively, based on diagnosis with microscopy at health centres and rapid diagnostic tests (RDTs) at health posts [3].

### Study design and sample size estimation

This study used a matched case–control design to investigate associations between malaria and individual/household risk factors. The study was undertaken as part of a larger cluster-randomized controlled trial (*MalTrials*) project that aimed to investigate whether the combination of LLINs with IRS will enhance the protective benefits of both interventions against malaria [17]. Each case was matched with two neighbourhood healthy controls based on age and same neighborhood or village. The matching was not done exactly on age. Instead the matching variable “age” was categorized into < 5 years old and with 10-year intervals (± 5 years) for those ≥ 5 years old.

Using two population proportions matched case–control sample size formula, the number of cases and controls required for the study was calculated assuming 95% confidence level, 80% power and 5% non-response rate with a 1:2 ratio of case to controls [21]. Taking household’s ownership of at least one LLIN as a major exposure variable associated with malaria, the probabilities of exposure among households of controls (Po) and cases (P1) were assumed to be 0.3 and 0.19, respectively. Finally, a minimum of 185 cases and 370 controls were estimated for the study. The calculated sample size was allocated to the study health centres and health posts based on the flow of malaria patients during the preceding months. The sample size was also assumed to adequately satisfy the analysis of the association between household’s ownership of LLINs and associated factors.

### Identification of cases and controls

Three health centres and nine health posts in the district were included in the study. Cases were defined as patients confirmed with malaria parasitaemia (*P. falciparum* or *P. vivax*) at the health posts or health centres using RDTs or microscopy, respectively. Cases were enrolled into the study consecutively from the health facilities until the allocated sample size was attained. Cases with signs of severity or from distant *kebeles* or areas were excluded.

Controls were healthy individuals selected from neighboring households nearest to the malaria cases and matched on age. They were considered to be healthy if they perceived healthy or the parents/guardians perceived the child to be healthy, and if they acknowledged no symptoms of malaria such as fever and had not been diagnosed and treated for malaria within a 2-week time prior to the presentation of the matched case at the health facility. However, they were not ascertained for malaria parasitaemia using either RDTs or microscopy due to financial and logistic constraints.

When more than two controls were available in the selected household, two were selected randomly. If there was no eligible control in the household, the next nearest neighboring household was selected. In this study, a household was defined as any unit headed by a male or female with his/her dependents who shared a common source of food and/or income and permanently resided in the area.

### Data collection

Data were collected between October 2014 and November 2015. Blood samples were collected from patients visiting health posts and health centers by the health workers as part of the routine malaria diagnosis and treatment. At the health posts, malaria diagnosis was made by the HEWs from blood samples collected from finger pricks using multi-species RDTs. The RDTs were the CareStart^®^ *Pf/Pv* combo test (Access Bio, Inc., Somerset, NJ, USA), which were individually packaged with an alcohol swab, lancet, capillary tube, buffer and test device. At health centres, thick and thin blood smears from finger pricks were collected by laboratory technicians following standard procedures and stained with Giemsa. Malaria parasitaemia was determined by examination of thick blood smears under oil immersion using a microscope. The *Plasmodium* species was determined on the thin blood smear, and anti-malarial treatment was performed following the national guidelines [3]. Artemether–lumefantrine (20/120 mg, Coartem^®^) and chloroquine (150 mg base) were given if RDT or microscopy was positive for *P. falciparum* or *P. vivax*, respectively.

Data on sex, age, result of malaria diagnosis, type of anti-malarial treatment and residential address were collected for each case identified at the health facilities using a logbook prepared for this study. Residential addresses were used to locate the cases. Trained field staff conducted a population-based house-to-house visits to follow-up the cases at their home in the community and find suitable neighborhood controls within a 2-week time from the date of diagnosis at the health facilities.

On finding the case and identifying the matching controls, information on demographics, housing, socio-economic characteristics and possible risk factors for malaria were collected using a pre-tested structured English questionnaire translated into the local *Afan Oromo* language. The head of each case and control household was interviewed about the knowledge of malaria, availability and number of LLINs in the household. If the presence of LLINs were reported, interviewers asked permission to enter the house, count and observe the nets. Finally, information on household assets such as radio, television, mobile telephone, refrigerator, table, chair, kerosene lamp, an electric stove or electricity, bicycle, motorcycle, cart, car, or land were collected to determine household wealth index.

### Statistical analysis

All questionnaires were manually checked for completeness and the data entered into a computer using SPSS version 23 (SPSS, Chicago, IL, USA). Descriptive analysis was conducted using SPSS, while all bivariate and multivariate analyses were performed using STATA version 13 (College Station, TX, USA). Bivariate and multivariate unconditional logistic regression models were used to calculate the crude odds ratios (COR) and adjusted odds ratios (AOR) with 95% confidence intervals (CIs) for any association between household’s ownership of LLINs and possible predictors. Ownership was defined as having at least one LLIN. A conditional logistic regression (CLR) model was performed to determine the association between the risk factor and malaria infection, using the clogit command in STATA [22].

In the bivariate analysis, independent variables that were associated with the dependent variable at *P* < 0.25 [22] were included in the multivariate regression model to identify those significant factors associated with the outcome variable, adjusting for other variables, and the level of significance was set at *P* < 0.05. Since the matching was done on “age” variable, this variable was not included in the multivariate CLR model for malaria risk assessment. The SURVEY (SVY) command in STATA was used to account for the clustered nature of the data with *kebele* as a primary sampling unit in all bivariate and multivariate models.

### Malaria knowledge score

Knowledge of malaria was defined based on the responses of the head of each case and control household to nine closed-ended questions on malaria. The knowledge score was constructed using “yes” and “no” responses: (1) Fever is the main symptom of malaria illness, (2) IRS kills mosquitoes, (3) IRS prevents malaria, (4) IRS is a chemical sprayed on the interior walls of a house, (5) IRS is an effective means for malaria prevention, (6) Re-plastering makes IRS ineffective, (7) Sleeping under LLINs every night prevents malaria, (8) Malaria is a preventable, and (9) treatable disease. Each question contributed one point to the overall knowledge score. The knowledge scores were finally categorized into binary exposure variable using the median score (low knowledge < median and high knowledge ≥ median) as a predictor for ownership of LLIN or malaria risk. The low levels of knowledge constituted 44% of the respondents while the high knowledge levels represented 56% of the study participants.

### Household wealth index

The relative wealth index was constructed for each household using the Principal Components Analysis (PCA) method in SPSS version 23 based on a combination of household and asset variables [23, 24]. Overall, 14 variables were subjected to PCA. However, variables such as ownership of bed and watch were given weights < 0.25 and excluded from the final model. The final PCA model for 12 items revealed the presence of three components with eigenvalues exceeding 1, explaining 21.81, 16.43, and 13.55% of the variance, respectively. The value of Keiser-Meyer-Olkin (KMO) measure of sampling adequacy was 0.651 and the Bartlett’s Test of Sphericity value reached statistical significance (*P* < 0.001). This method gave the greatest weight to ownership of livestock, herds, or farm animal (0.829), land used for agriculture (0.793), ownership of mobile phone (0.608), number of sleeping areas in the household (0.552), ownership of bank account (0.550), radio (0.540), television (0.475), number of rooms (0.466), electricity (0.434), ownership of bicycle (0.356) and cart (0.321), and separate kitchen (0.291). Finally, the households were categorized into three socio-economic levels based on the PCA scores: lower (poorest) (33.3%), middle (33.8%) and upper (least poor) (32.9%).

### Ethical considerations

The study was approved by the Institutional Review Board of the College of Health Sciences of Addis Ababa University and the National Ethical Committee of the Federal Ministry of Science and Technology in Ethiopia as part of a larger cluster-randomized controlled trial that aimed to investigate whether the combination of LLINs with IRS will enhance the protective benefits of both interventions against malaria [17]. All cases and controls including their households were informed about the purpose of the study, data collection procedures, potential benefits and harms, and confidentiality issues. Oral informed consent was obtained from all older children and adults, while verbal assent was obtained from the parents or caregivers/guardians of young children (< 15 years old). Cases were treated for malaria as per the national guidelines [3].

## Results

### Characteristics of the respondents and households

A total of 191 case and 377 control households were included in the main analysis of the data. Six cases and 14 controls were excluded from the analysis due to incomplete information or matching problems. Table 1 shows characteristics of the respondents (or household heads) and their households included in the study. More than 98% of the respondents were males, with the majority (77.4%) being between 25 and 44 years of age. The majority were married (96.8%), Muslims (91%), Oromo ethnic group (98.4%) and farmers (90.3%). About 55% of the respondents replied that they could read and write. The average household size of the cases and controls was 4.6 per case household and 4.4 per control household. By household wealth index, there was no difference between case and control households.

**Table 1:** Characteristics of the respondents and their households among case and control households

Characteristics	Total, n (%)	Cases, n (%)	Control, n (%)	P value
Sex				
Male	561 (98.8)	188 (98.4)	373 (98.9)	0.603
Female	7 (1.2)	3 (1.6)	4 (1.1)	
Age (years) groups				
18–24	42 (7.4)	17 (8.9)	25 (6.6)	
25–34	244 (43.0)	75 (39.3)	169 (44.8)	0.654
35–44	193 (34.0)	70 (36.6)	123 (32.6)	
45–54	67 (11.8)	22 (11.5)	45 (11.9)	
≥ 55	22 (3.9)	7 (3.7)	15 (4.0)	
Marital status				
Married	550 (96.8)	184 (96.3)	366 (97.1)	0.631
Other	18 (3.2)	7 (3.7)	11 (2.9)	
Religion				
Muslim	517 (91.0)	170 (89.0)	347 (92.0)	0.232
Christian	51 (9.0)	21 (11.0)	30 (8.0)	
Ethnicity				
Oromo	559 (98.4)	188 (98.4)	371 (98.4)	0.985
Other	9 (1.6)	3 (1.6)	6 (1.6)	
Educational status				
No education	162 (28.5)	52 (27.2)	110 (29.2)	
Read and write	319 (56.2)	113 (59.2)	206 (54.6)	0.555
Primary (grade 1–6) or higher	87 (15.3)	26 (13.6)	61 (16.2)	
Occupation				
Farmer	513 (90.3)	172 (90.1)	341 (90.5)	0.879
Other	55 (9.7)	19 (9.9)	36 (9.5)	
Household size				
< 5	293 (51.6)	90 (47.1)	203 (53.8)	0.130
≥ 5	275 (48.4)	101 (52.9)	174 (46.2)	
Household wall structure				
Wood only	60 (10.6)	23 (12.0)	37 (9.8)	
Wood with mud	464 (81.7)	148 (77.5)	316 (83.8)	0.137
Wood with mud and cement	44 (7.7)	20 (10.5)	24 (6.4)	
Household roof structure				
Corrugated iron	200 (35.2)	69 (36.1)	131 (34.7)	0.745
Thatched	368 (64.8)	122 (63.9)	246 (65.3)	
Toilet facility				
Pit latrine	503 (88.6)	168 (88.0)	335 (88.9)	
VIP/flush latrine	39 (6.9)	14 (7.3)	25 (6.6)	0.945
No latrine	26 (4.6)	9 (4.7)	17 (4.5)	
Household wealth index				
Lower (very poor)	189 (33.3)	63 (33.0)	126 (33.4)	
Middle	192 (33.8)	65 (34.0)	127 (33.7)	0.994
Higher (least poor)	187 (32.9)	63 (33.0)	124 (32.9)	
N	568	191	377	

### Characteristics of the cases and controls

A total of 191 cases of malaria and 377 controls were included in the final data analysis. Cases were identified from 27 *kebeles* and the number varied from three to 14. All cases were traced and nobody or household from the cases or controls refused to take part in the study. About 52% (n = 109) of the cases were identified at the health centres, while 42.9% (n = 82) cases were identified from the health posts. All cases were infections due to either *P. falciparum* (71.2%, n = 136) or *P. vivax* (28.8%, n = 55). About 45% (n = 85) and 48.3% (n = 182) were males among cases and controls, respectively. The mean (± standard deviation) age was 15.46 (± 11.5) years for cases and 15.37 (± 11.8) years for controls. About 35% (n = 66) cases and 38.5% (n = 145) controls were less than 10 years of age.

### Household’s ownership of LLINs and its association with other factors

About 31% (n = 174) of the total interviewed households (29.8% case households and 31% control households, *P* = 0.77) had at least one functional LLINs that could be used while sleeping and almost all nets were PermaNet^®^ 2.0. From the total net owning households, a total of 341 LLINs were identified, where 30% (n = 52) of the households had one net, 46% (n = 80) had two, and 24% (n = 42) had three or more nets. Only 24.3% (n = 138) of the total respondents reported that they slept under LLINs the previous night. Particularly, 16.4% (n = 93) of the respondents reported they always or frequently slept under a net whilst 13.4% (n = 76) reported they occasionally slept under nets.

Based on the bivariate analysis, a strong association was found between ownership of LLINs and educational status of the head of household, knowledge about malaria (COR = 1.97, 95% CI 1.12–3.48), number of sleeping spaces in the household and household wealth index (Table 2). Households with respondents who could read and write or with primary or higher education were more likely to own LLINs. In addition, households with corrugated iron roofs were associated with increased ownership of LLINs (COR = 2.67, 95% CI 1.37–5.21). Similarly, households in the higher wealth quantile were more likely to own LLINs compared to households in the lower wealth category.

**Table 2:** Individual and household factors associated with ownership of LLINs in the bivariate analysis

Characteristics of the household	Household owned at least one LLIN		COR (95% CI)	P value
	Yes, n (%)	No, n (%)		
Educational status of the respondent				
No education	13 (7.5)	149 (37.4)	1	
Read and write	135 (77.6)	184 (46.7)	8.40 (2.42, 29.18)	0.002
Primary (grade 1–6) or higher	26 (14.9)	61 (15.5)	4.88 (1.60, 14.93)	0.007
Occupation of the respondent				
Farmer	150 (86.2)	363 (92.1)	0.53 (0.18, 1.56)	0.242
Other	24 (13.8)	31 (7.9)	1	
Respondent’s knowledge on malaria				
High	117 (67.2)	201 (51.0)	1.97 (1.12, 3.48)	0.021
Low	57 (32.8)	193 (49.0)	1	
Household size				
< 5	78 (44.8)	215 (54.6)	0.68 (0.36, 1.26)	0.207
≥ 5	96 (55.2)	179 (45.4)	1	
Households with				
Cases	57 (32.7)	134 (34.0)	0.95 (0.79, 1.14)	0.537
Controls	117 (67.3)	260 (66.0)	1	
Number of sleeping areas in the household				
1	18 (10.3)	127 (32.2)	1	
2	61 (35.1)	181 (45.9)	2.69 (0.96, 7.59)	0.060
≥ 3	95 (54.6)	86 (21.8)	2.21 (0.78, 6.29)	0.132
Household roof structure				
Corrugated iron	89 (51.1)	111 (28.2)	2.67 (1.37, 5.21)	0.006
Thatched	85 (48.9)	283 (71.8)	1	
House sprayed with insecticide				
Yes	112 (64.4)	215 (54.6)	1.50 (0.36, 6.27)	0.562
No	62 (35.6)	179 (45.4)	1	
Household wealth index				
Lower (very poor)	28 (16.1)	161 (40.9)	1	
Middle	68 (39.1)	124 (31.5)	3.15 (1.60, 6.21)	0.002
Higher (least poor)	78 (44.8)	109 (27.7)	4.11 (2.22, 7.64)	< 0.001
N	174	394		

*LLINs* long-lasting insecticidal nets, *COR* crude odds ratio, *CI* confidence interval

In the final multivariate model, five of the seven explanatory variables including respondent’s educational status, farming occupation (AOR = 0.35, 95% CI 0.17–0.76), higher knowledge about malaria (AOR = 2.47, 95% CI 1.44–4.22), and number of sleeping areas in the household and corrugated iron roof (AOR = 2.49, 95% CI 1.36–4.58) were significantly associated with ownership of LLINs after adjusting for all other explanatory variables in the model (Table 3). Households with respondents who could read and write or with primary/higher education showed more than fivefold higher odds of LLINs ownership. Households with ≥ 3 sleeping areas had significantly higher odds of LLINs ownership (AOR = 6.71, 95% CI 2.40–18.77). However, no significant association was found between household wealth index and LLINs ownership after adjusting for other explanatory variables in the final multivariate model.

**Table 3:** Individual and household factors associated with ownership of LLINs in the multivariate analysis

Characteristics of the household	AOR (95% CI)	P value
Educational status of the respondent		
No education	1	
Read and write	6.88 (2.30, 20.55)	0.001
Primary (grade 1–6) or higher	5.40 (1.57, 18.55)	0.009
Occupation of the respondent		
Farmer	0.35 (0.17, 0.76)	0.009
Other	1	
Respondent’s knowledge on malaria		
High	2.47 (1.44, 4.22)	0.002
Low	1	
Household size		
< 5	0.78 (0.45, 1.20)	0.209
≥ 5	1	
Number of sleeping areas in the household		
1	1	
2	1.77 (0.53, 5.87)	0.335
≥ 3	6.71 (2.40, 18.77)	0.001
Household roof structure		
Corrugated iron	2.49 (1.36, 4.58)	0.005
Thatched	1	
Household wealth index		
Lower (very poor)	1	
Middle	1.37 (0.51, 3.65)	0.520
Higher (least poor)	0.80 (0.43, 1.50)	0.473

*AOR* adjusted odds ratio, *CI* confidence interval

### Association between malaria infection and other factors

Table 4 shows individual and household risk factors associated with malaria infection in the bivariate matched analysis. Among the 15 independent individual and household variables assessed in the matched bivariate analysis, none of them were significantly associated with malaria infection. Case households were more likely to have family size of ≥ 5 but not statistically significant (COR = 1.57, 95% CI 0.99–2.48). A reported history of staying overnight outdoor in the previous month (COR = 1.91, 95% CI 0.94–3.88) and lack of reporting mosquito biting during the last month (COR = 0.36, 95% CI 0.11–1.11) were associated with malaria infection, but not statistically significant. Sex and other variables with *P* < 0.25 in the matched bivariate analysis were included in the multivariate CLR model (Table 5). However, none of the variables included in the model were statistically significantly associated with malaria infection after adjustment for all other variables except history of mosquito biting during the last month with borderline statistical significance (AOR = 0.33, 95% CI 0.11–1.04).

**Table 4:** Individual and household risk factors associated with malaria infection in the matched bivariate analysis

Characteristics	Cases, n (%)	Control, n (%)	COR (95% CI)	P value
Sex				
Female	106 (55.5)	195 (51.7)	1	
Male	85 (44.5)	182 (48.3)	0.82 (0.50, 1.35)	0.413
Household size				
< 5	90 (47.1)	203 (53.8)	1	
≥ 5	101 (52.9)	174 (46.2)	1.57 (0.99, 2.48)	0.055
Household wall structure				
Wood only	23 (12.0)	37 (9.8)	1	
Wood with mud	148 (77.5)	316 (83.8)	0.62 (0.26, 1.44)	0.252
Wood with mud and cement	20 (10.5)	24 (6.4)	1.41 (0.65, 3.10)	0.374
Household roof structure				
Corrugated iron	69 (36.1)	131 (34.7)	1	
Thatched	122 (63.9)	246 (65.3)	0.89 (0.43, 1.84)	0.735
Keep livestock inside house				
No	183 (95.8)	368 (97.6)	1	
Yes	8 (4.2)	9 (2.4)	1.62 (0.64, 4.13)	0.296
Opening windows on retiring to sleep				
No	179 (93.7)	361 (95.8)	1	
Yes	12 (6.3)	16 (4.2)	1.83 (0.72, 4.66)	0.196
Frequency of mosquito biting during the last month				
Frequently	12 (6.3)	12 (3.2)	1	
Occasionally	115 (60.2)	193 (51.2)	0.83 (0.35, 1.98)	0.662
Not at all	64 (33.5)	172 (45.6)	0.36 (0.11, 1.11)	0.073
Stayed overnight outdoor in the last month				
No	166 (86.9)	343 (91.8)	1	
Yes	25 (13.1)	31 (8.2)	1.91 (0.94, 3.88)	0.073
Household owned at least one LLIN				
No	134 (70.2)	260 (69.0)	1	
Yes	57 (29.8)	117 (31.0)	0.82 (0.37, 1.80)	0.602
No. of LLIN owned by household				
0	134 (70.2)	260 (69.0)	1.15 (0.45, 2.91)	0.766
1	16 (8.4)	36 (9.6)	0.86 (0.31, 2.39)	0.768
≥ 2	41 (21.5)	81 (21.5)	1	
Anyone in the household slept under LLIN last night				
No	135 (70.7)	265 (70.3)	1	
Yes	56 (29.3)	112 (29.7)	0.95 (0.44, 2.03)	0.889
Had malaria during the last 6 months				
No	159 (83.3)	326 (86.5)	1	
Yes	32 (16.8)	51 (13.5)	1.53 (0.45, 5.22)	0.487
House sprayed with insecticide				
No	81 (42.4)	160 (42.4)	1	
Yes	110 (57.6)	217 (57.7)	0.96 (0.32, 2.87)	0.945
Respondent’s knowledge on malaria				
Low	80 (41.9)	170 (45.1)	1	
High	111 (58.1)	207 (54.9)	1.21 (0.74, 1.95)	0.434
Household wealth index				
Lower (very poor)	63 (33.0)	126 (33.4)	1	
Middle	65 (34.0)	127 (33.7)	1.01 (0.66, 1.55)	0.953
Higher (least poor)	63 (33.0)	124 (32.9)	1.01 (0.62, 1.70)	0.921
N	191	377		

*COR* crude odds ratio, *CI* confidence interval, *LLINs* long-lasting insecticidal nets

**Table 5:** Individual and household risk factors associated with malaria infection in the matched multivariate analysis

Characteristics	AOR (95% CI)	P value
Sex		
Female	1	
Male	0.88 (0.52, 1.48)	0.607
Household size		
< 5	1	
≥ 5	1.48 (0.91, 2.39)	0.109
Keep livestock inside house		
No	1	
Yes	1.43 (0.83, 2.49)	0.190
Opening windows on retiring to sleep		
No	1	
Yes	1.26 (0.51, 3.11)	0.597
Frequency of mosquito biting during the last month		
Frequently	1	
Occasionally	0.77 (0.31, 1.94)	0.565
Not at all	0.33 (0.11, 1.04)	0.058
Stayed overnight outdoor in the last month		
No	1	
Yes	1.57 (0.76, 3.24)	0.209
Had malaria during the last 6 months		
No	1	
Yes	1.09 (0.27, 4.44)	0.904
N		

*AOR* adjusted odds ratio, *CI* confidence interval

## Discussion

This study showed that only 31% of households owned at least one LLIN, and 21.5% of the net owning households had at least two nets. Educational status of the heads of households, occupation, knowledge about malaria, type of roof and number of sleeping areas in the household were independent predictors of ownership of LLINs. No statistically significant association with malaria infection was found in this study with regard to sex, household size, keeping livestock in the household, opening windows on retiring to sleep, history of malaria illness during the last 6 months, and staying overnight outdoor in the last month.

In the current study area, ownership of LLINs was very low despite efforts towards the universal net coverage by the Ministry of Health with free mass distribution and replacement of worn out nets through routine distribution [15]. The Ethiopian malaria prevention and control strategy aims to achieve universal coverage of all households at risk of malaria with a minimum of one net per two persons [15]. A study undertaken in the current study area in 2013 showed 27% ownership of LLINs by households [20]. One potential reason for the low level of LLINs ownership could be seasonality and low prevalence of malaria since people tend to own and use LLINs less likely during low transmission season [25]. Several studies demonstrated low prevalence and incidence of malaria in Ethiopia [5, 6, 20].

In this study, household heads higher educational level was associated with ownership of LLINs. Studies have shown that higher educational level and malaria knowledge positively influencing ownership of nets by households [10, 26–28]. This could be due to the fact that more educated and knowledgeable people were able to access information about the importance and benefits of mosquito nets, leading to better practices to protect and retain the received nets.

In the current study, household wealth index was not statistically associated with ownership of LLINs. A study conducted in south-central Ethiopia [29] reported similar findings. One possible explanation for the lack of association between LLINs ownership and socio-economic level would be that individuals do not buy additional nets for their households and hence are more likely to depend on free distribution or replacement of LLINs. In contrast, higher ownership of LLINs amongst households with higher socio-economic status has been observed previously in Ethiopia [30] and Tanzania [28].

According to the 2014/15 national malaria data, *P. falciparum* accounted for about 64% of all malaria cases, followed by *P. vivax* (about 36%) [7], whilst *P. falciparum* represented about 77% of confirmed cases in the 2011 EMIS report [4]. All malaria infections identified in this study were due to either *P. falciparum* (71%) or *P. vivax* (29%). A health facility-based study conducted in 2012 in the same geographic zone showed that *P. vivax* and *P. falciparum* accounted for 57 and 41% of cases, respectively [31]. However, in a longitudinal community-based study conducted in 2013, *P. vivax* (85%, n = 33) outweighed *P. falciparum* (15%, n = 6) although the overall number of confirmed cases was small [20]. Heterogeneity between and within villages has previously been reported and is particularly a feature of malaria epidemiology in Ethiopia [32–35].

The findings of the current study show lack of association between malaria risk and household factors such as house structure, keeping livestock inside house, and opening windows on retiring to bed. This is consistent with another study in south-central Ethiopia that showed lack of association between housing structure and malaria incidence [14]. Another study in Yemen reported lack of association between open window and malaria risk [36]. In contrast, opening windows when retiring to bed or the presence of openings in the walls during peak transmission season was strongly associated with increased risk of malaria in South Africa [37] and, therefore, closing windows or openings in the walls can act as a protective barrier to prevent mosquitoes from entering houses.

The study findings show lack of association between ownership of LLINs and risk of malaria, which is probably due to the similar level of net coverage among case and control households. Another possible explanation for this lack of association can be attributed to low usage of nets in the area due to low transmission of malaria coupled with relatively high IRS coverage. A study in South Africa showed a lack of association between malaria risk and ownership of nets [37]. However, a study in Tanzania has demonstrated strong association between higher community LLINs coverage and reduced risk of malaria [38].

In the current study, respondent’s knowledge about malaria was not statistically significantly associated with malaria risk. The possible explanation that could be put forward is that the level of knowledge among the cases and controls was similar. However, it is likely to argue that increased level of knowledge on malaria is associated with reduced risk of malaria. One possible explanation could be that people who have a high level of knowledge are in a better position to protect themselves against malaria.

It is clear that household socio-economic status plays an important role in malaria prevention because people with higher income have more resources to access personal protective measures against malaria. However, in the present study it could not be determined whether households in the lower wealth category were at increased risk of malaria, consistent with other studies that reported lack of association between household wealth and malaria risk [14, 39]. In contrast, a case–control study undertaken in South Africa showed that households in the higher wealth categories had a lower risk of malaria than households in the poorer categories [37]. This is also consistent with other studies that showed strong association between higher household socio-economic status and reduced risk of malaria [12, 38, 40].

Several potential limitations may affect the findings of this study. The study was based on malaria cases identified at health facilities, controls were not tested for malaria, the findings may be affected by three sources of bias (selection, recall and information bias), and the study did not evaluate a key outcome of LLINs use. This study used a neighbouring household as a control and the selected households might have the same background variables as the cases. This could explain some of the findings, for example, that there was no association between malaria infection and wealth. Furthermore, almost all the study participants for assessment of the knowledge on malaria were male respondents and may not reflect the women’s perspective. Finally, given that this study was conducted in a low transmission setting with low LLINs coverage, the findings may not be generalizable to other settings with high malaria incidence. However, as the aim of the study was to identify potential factors associated with ownership of LLINs and malaria infection, the findings could still serve as an important input to inform proper targeting of anti-malarial interventions.

## Conclusions

Household ownership of at least one LLIN was very low and it is far from being universal. This study confirmed that educational status, occupation, knowledge about malaria and house structure were important predictors of ownership of LLINs. This study warrants a population-based study to identify effective measures by which universal household ownership of LLINs can be achieved and sustained. The importance of individual and household risk factors associated with malaria infection was less evident in the present study in comparison with similar studies reported elsewhere. Further studies are needed to generate more evidences, particularly in low transmission settings similar to the current study area.
